# Health perceptions among victims in post-accord Colombia: Focus groups in a province affected by the armed conflict

**DOI:** 10.1371/journal.pone.0264684

**Published:** 2022-03-02

**Authors:** Catalina González-Uribe, Antonio Olmos-Pinzón, Sebastián León-Giraldo, Oscar Bernal, Rodrigo Moreno-Serra

**Affiliations:** 1 School of Medicine, Universidad de los Andes, Bogotá, Colombia; 2 Alberto Lleras Camargo School of Government, Universidad de los Andes, Bogotá, Colombia; 3 Interdisciplinary Centre of Development Studies, Universidad de los Andes, Bogotá, Colombia; 4 Centre for Health Economics, University of York, York, United Kingdom; Queen’s University at Kingston, CANADA

## Abstract

The peace agreement with the Colombian guerrilla group Fuerzas Armadas Revolucionarias de Colombia—Ejército del Pueblo represented an opportunity for peacebuilding and victims’ reparation, rather than the end of the internal armed conflict. In this context, this study aimed to uncover the consequences of conflict on victims’ health and on health service provision, and their perceived health status during the post-accord stage in the Meta region, located in the country’s eastern plains. Historically, this region has been one of the territories most affected by the presence of conflict-related groups and armed confrontations. Through focus groups, this research explored the health perceptions and experiences of victims of armed conflict. Ten focus groups were conducted with men and women, victims of the armed-conflict, in four municipalities with different degrees of armed conflict intensity. The focus group transcripts were coded using NVivo. The results show that the way women have experienced conflict and the effects of conflict on mental health in general for men, women, and children were recurrent themes in the dialogue of victims. Likewise, it highlights the need to understand the barriers that the current health model imposes on the right to health itself. From the victim’s perspective, they experience stigmatization, discrimination, and revictimization when accessing health services. These barriers co-occur along with structural limitations of the health system that affect the general population.

## Introduction

The Colombian armed conflict has affected the lives of millions of people in the country. It is estimated under the Victim´s Law that more than eight million people suffered individually or collectively violations of International Humanitarian Law or other grave violations of international human rights norms since 1 January 1985 [1]. The vast majority, 7,585,536 people, were forced from their homes [2], followed by homicides, threats, enforced disappearances, loss of property, terrorist acts, kidnappings, sexual violence, damage from explosive devices, among others [3]. These direct and indirect impacts on people’s physical and mental health, require state policies at national and regional levels. Hence, the National Health Plan 2012–2021 [4] has already recognized that the victims of the armed conflict are a special interest group for policies in Colombia that seek to reduce health inequities.

The peace agreement signed in 2016, between the Fuerzas Armadas Revolucionarias de Colombia—Ejército del Pueblo FARC-EP, the oldest guerrilla group in the country, and the national government created expectations about possible changes in the region. There was a generalized hope that the state would increase its presence in territories with a historical presence of armed groups. It was also expected that people’s living conditions would improve through improvements in health and social services.

In the Peace Accord, health was not included as a separate theme, but, instead, was considered in two of the six points of the final agreement [5]. The first of the key points, the *Comprehensive Rural Reform*, refers to health as a fundamental aspect for creating well-being and quality living conditions for the rural population. On the other hand, in the fifth point of the agreement, *Justice for Victims*, the importance of psychosocial care and victim rehabilitation was emphasized (section 5.1.3.4 of the Peace Agreement). Additionally, the implementation of Law 1448 of 2011 (known as the "Victims’ Law") continued, prioritizing victims’ psychosocial and healthcare well-being.

On the other hand, the current health service model is insufficient to meet the health needs of the general population [6–8], much less those of the most vulnerable populations, including the victims of the conflict and those who live in rural areas. Additionally, the Peace Agreement did not mean the end of violence in Colombia, although it decreased during the negotiation period, attacks and killings of social leaders and human rights defenders have increased since the signing of the final agreement [9].

Thus, post-accord Colombia represents a complex setting because violence and structural problems persist throughout the country. Nevertheless, it also represents an opportunity to reduce inequities in social services as part of peacebuilding. This period is a crucial moment to supply the, previously unmet, health care needs of the population where the FARC-EP had a strong presence. Survivors of violence must be at the center of the public agenda and deserve dignified and timely attention. If the state does not fill these gaps, the country’s public health situation may worsen, considering additional stressors such as the recent health emergency generated by Covid-19 pandemic.

In this context, discussions on reparations of victims become relevant. Reparation refers to the set of measures aimed at restoring the rights and improving the living conditions of those who have been victims of human rights violations and promoting policies that guarantee non-repetition [10]. In Colombia, reparation is understood as a process that seeks to dignify victims through measures that alleviate their suffering, compensate for the social, moral, and material losses they have suffered, and restore their civil rights [11]. The study of Rettberg and Prieto [12] showed that most victims prioritize material reparations but obtaining some guarantee that they will not become victims again is also one of their main concerns.

The studies that have contributed to the discussion on reparations for victims from the health perspective are varied. Previous qualitative work has studied the dynamics of resettlement of families displaced by the conflict [13, 14] and their access to health services [15]; the mental health situation and challenges in post-conflict contexts [16–19]; health as a right in post-conflict settings [20]; the main sexual and reproductive health conditions of women victims of the conflict [21]; and women’s views on the impact of forced displacement on health [22]. Most of these studies recognize the resilience of the conflict’s victims and point to the need of social policies that move beyond humanitarian assistance. Qualitative studies on mental health include the psychosocial profile of the victims and associated factors [23–25]; and mental health interventions and required care for this population [26–28].

### Victims, trauma, and health

For our analysis, the category of "victim" is understood beyond the victimizing event. We understand this category along a victim-witness-political actor continuum. The subject who narrates his experiences of violence does so with a political agency that allows him to activate demands for restoration and truth [29]. Unlike the patient category, where the subject’s problems are not necessarily experienced collectively, the victim category refers to those who claim justice to heal suffering produced by societal trauma [26]. When victims speak out, a piece of social fabric is formed between those who speak and those who listen. This fabric feeds common actions and finds a public ethic of recognition, based on what Jimeno et al. [30] called "emotional communities" [31]. In this sense, the victim is a political category used to communicate and negotiate with institutions, where claims for truth, justice, material reparation, and support are processed. Thus, victims of violence are conceived as right-bearers and objects of public policy and active political subjects. This victim’s conception implies an understanding that the subjects’ narratives account for traumatic events but also support civil actions.

On the one hand, the label of victims is an instrument of identity reconstruction [32], and, on the other, they constitute an appropriation of suffering for political uses [33]. The victim’s figure as a witness allows a move from the particular subjective terrain towards the shared field and broad audience in this framework. This process allows the suffering of others to be assumed as their own, followed by solidarity that can allow reparations and avoid repetitive traumatic events [34].

In this process, historical trauma becomes relevant, as complex and collective trauma experienced through time from generation to generation by a group of people who share an identity, affiliation, or circumstance [35]. It connects personal stories of traumatic events with current experiences and contexts, including the current health of a group or community. The connection between historical trauma and recent experiences, related narratives, and health impacts can function as a source of anguish and resilience [36]. In this sense, the victims’ narratives link the experience of traumatic events with their current health. This happens by interpreting and communicating the link between stress and disease and the various levels at which life factors affect health and disease [37]. Historical trauma’s potential allows one not to fall into causal models but rather to identify how current experiences and their narratives are connected to a particular group [36].

Likewise, it is important to account for the victims’ health experiences; not only with regard to their individual perceptions but to the social processes and mechanisms by which victims connect their subjective experiences when they talk about health. We understand the experience of health as a social process of production of meaning. This process includes the intersubjective interpretation of disease, pain, life, medicalization scenarios, quality of life and health as capital, right, and duty [38].

Therefore, this study explores victims’ health perceptions and experiences with service provision in the province of Meta, one of the territories most affected by the country’s armed conflict. Our study enquires about the following topics: What are the victim’s health perceptions and experiences with health services? How has conflict affected their perceived health status and access to health provision? The analysis of the results sheds light on the problems that victims experience in post-accord Colombia. We recognize that victims’ health and healthcare problems also correspond to the problems faced by other Colombians. However, we are interested in the victims’ particular perceptions regarding health and their need for differential attention as political subjects. As members of vulnerable populations, perceptions and experiences of victims must be heard in the process of restoring the country’s civil rights. This research took place in 2019, three years after the peace accord was signed. The results offer a glimpse of the early period of implementation.

## Methodology

### Research methodology and data collection method

This is a qualitative study with a constructivist approach. Participants are viewed as creators of their social world, and the researchers interpret it subjectively [39]. We used a focus group study technique to generate data from the free-flowing interaction, not just by ask-answer questions [40]. The target ´population for our focus groups was the victims of each municipality. Considering the topics discussed, efforts were made to convene separate and mixed focus groups of women and men. Participants voluntarily attended the call, and most of them were women. Nine focus groups were organized with victims in four municipalities with different degrees of armed conflict intensity, according to the typology proposed by the Resource Center for Conflict Analysis (CERAC), as summarized in Table 1.

**Table 1:** Focus groups by location, gender and number of participants.

Focus Group	Place	Men	Women
I	Villavicencio (capital city strongly affected)	0	8
II		12	0
III		0	10
IV	Castilla La Nueva (without conflict)	0	6
V		4	4
VI	Granada (lightly affected)	0	6
VII		0	5
VIII		0	7
IX	Vistahermosa (strongly affected)	7	0
X		0	6
**TOTAL**		23	52

### Recruitment, participants, and focus group procedure

The participants’ recruitment was conducted by contacting local organizations of victims in four municipalities of the region of Meta, Colombia. Participants were contacted mainly through leaders within organizations. All participants were adults and participated in sessions where an informed consent was read and discussed to answer any questions about the purpose of the study. Our nine focus groups were held in closed spaces in each municipality during a one-week period in September 2019. Each focus group lasted approximately one hour and was moderated by research assistants, using a semi-structured guideline. Each focus group began with an initial round in which all the participants and moderators introduced themselves. The moderators guided the discussion following a questioning route, first asking how participants defined and understood health. Then, key topics were explored, such as the impacts of conflict on participants and general community mental and physical health, experiences of using health services, strategies to solve health problems, changes after the peace agreement, and recommendations about specialized care for victims. Also, moderators encouraged the participants to speak freely about other related themes and openly share their views. The recordings of the nine focus groups were transcribed and anonymized by replacing participants’ names with pseudonyms.

### Data analysis

The transcriptions were analyzed using the NVivo qualitative data analysis software, following a thematic analysis [41]. First, an initial set of codes was generated based on the first reading of transcriptions and predefined themes of interest. Then, the codes were organized systematically by establishing initial family codes. New codes arose related to themes of interest during the initial codification process of the first three focus groups. At this point, we checked if the collected data in each code worked with code families and reassign codes in the correct code family if necessary, considering internal homogeneity and external heterogeneity (Patton’s, 1990 in Braun and Clarke, 2014). Once we coded all the transcriptions, we merged codes with similar data and less frequency and selected extracts from the transcriptions for all predefined and emergent codes. As a result of the codification stage, we created an extensive report organized by general themes. In doing this, we described the information in a concise, coherent, logical, non-repetitive, and representative way across all themes.

### Ethical considerations

The Ethical Review Committee of Universidad de los Andes approved the study. Participants were informed of their right to decline or withdraw from the study at any time without any adverse consequences. An informed consent was obtained from each participant, and the investigators committed to safeguarding the confidentiality and anonymity of the data collected. Participants did not receive any financial compensation for taking part in the study. All costs associated to their participation in the study, like transportation to the interview site were covered by the research team.

All transcriptions were conducted by a specialized third party who agreed to and signed confidentiality agreements to guarantee anonymity and reduce biased interpretations. All researchers in the project had access to these transcriptions once they were anonymized. Researchers who conducted the fieldwork had additional mentoring about qualitative methodology, including ethical considerations, and received voluntary psychological support after the fieldwork to account for the personal challenges of conducting this research.

## Results

### Meanings of health

The opening question of our focus groups was designed to start by exploring our participants’ definitions of health. This allowed us to consider the meanings that the participants associated with health when mentioning and hearing that word. It also allowed us to consider conceptions and subjective factors that inform participants’ health perceptions as integral to individuals’ and groups’ general well-being [42].

The responses on the meaning of health among focus group participants were varied. Some mentioned health as an integral component of the human being, including physical, mental, emotional, and spiritual elements. Similarly, others referred to health as a way to achieve autonomy in lifestyle choice. Others, instead, equated health to the provision of health services or a constitutional right. Thus, while some ideas were related to the concept of human well-being, others referred to the right to health and health care. The latter was accompanied by a series of negative perceptions of the healthcare system. Two participants explained why, for some, the word health has more to do with the system than with people’s well-being. For them, although they agree that health should be understood as a state of human well-being, they still tend to think, when asked about the meaning of health, about the problems they face when accessing health services:

*Woman:* *when you ask us about health*, *we think about those shortcomings that health care has*, *but health is the physical and mental state of each human being*, *being in good condition*, *physically*, *mentally*, *and psychologically*. *When they ask us about health*, *we immediately associate it with the whole problem that we have with healthcare*.

*Moderator:* *And why do you think that happens?*

*Woman:* *because we have a negligent state and all the health resources have been stolen from it*, *that’s why we now suffer the consequences of poor healthcare*.

*Woman (b):* *because by the culture*, *we are rooted in the health problem*, *so when you ask us*, *what is health*? *We forget that it is an optimal physical*, *mental*, *and psychological state because we even forgot that we should be well*. *After all*, *it has become normal for us to be sick or not to be treated or not get what we need to have a decent life*. *(Castilla la Nueva_sept3 women’s focus group)*

This dual conception of health continued to appear in the other topics that were discussed during the focus groups. As will be seen in the discussion, it is a notion that embodies victims’ experiences and perceptions regarding their health.

### Effects of the armed conflict on victims’ health

The effects of the armed conflict on people’s health were varied, as the participants pointed out. They reported bomb explosions, gunshot wounds, death threats, and sexual violence. Additionally, it was mentioned that the aerial spraying of glyphosate caused respiratory problems and skin diseases. Several participants mentioned that some of their children suffered traumatic events when they were young, which continued to have sequelae—both physical and emotional—during their lives. This section will delve into the two topics most discussed in the focus groups: how women have experienced conflict and the effects of conflict on mental health for children, women, and men. These were topics that participants were more openly discussed and where they found common ground.

### Multiple burdens for women in conflict-affected contexts

In the six focus groups held with women, they discussed how women had experienced some of the most painful and impactful effects of war gender-based violence; becoming the head of the family and primary breadwinner; receiving all kinds of stressors in the domestic sphere and having difficulties when reporting events of violence and revictimization while accessing health services. The type of violence most mentioned was the sexual violence suffered at the hands of members of illegal armed groups. Among these testimonies were women who suffered this type of violence while undertaking searches for children kidnapped by the guerrillas.

Most women affirmed that they have not received healthcare from the state or that this care has not had the desired effects. Thus, they demanded that more attention be paid to this issue, so victims can better cope with the trauma of war. Besides, psychological attention often leads to reliving wounds left by the war, which sequentially leads to discontinuation of care.

*Women:* *But if we had psychologists at the beginning*, *things would have been different*.

*Women (b):* *Because we were*, *for a time*, *very abandoned by the state*.

*Women:* *Because with my mom—I was 11 years old when we suffered the loss of my brother—we have never had a psychologist; we have overcome it ourselves*. *See here most are very few*, *from 10–15 years that we have already been in the conflict*, *and just now we are being seen by a psychologist*, *and I say*: *what for now*? *(Focus group with women in Vista Hermosa Sept5)*

However, there was also a call to improve how women report acts of violence or trauma. As reported by participants, some aspects make it difficult for women victims of sexual violence to report trauma. The first is shame or fear of being judged. The second aspect is often having to tell strangers, and in the worst case, when they are men. Also, another difficulty is the fact of having to repeat the same story over and over again, which relives traumatic events:

*Woman:* *Well*, *on the issue of women*, *I think the issue of sexual violence has been problematic*. *Because a woman becomes a victim of sexual violence*, *she has to tell everyone what happened*, *and we as victims if we feel embarrassed to talk about what happened to us at home*, *much less to several unknown people (*…*) and having to repeat the same story and go from one place to another*. *To the doctor*, *to the police station*, *to the Family Commissioner*, *to the Prosecutor’s Office*, *the route’s whole process is like a big mess*. *(Focus group women Vista Hermosa Sept5)*

Added to this context, the areas most affected by the conflict have higher fertility rates, poor access to obstetric care, and poor knowledge of STIs [21], which further complicates the consequences of gender violence.

On the other hand, men who participated in the focus groups did not tend to speak about their individual experiences of violence personally (being victims of the armed conflict), except for some who mentioned direct threats. Instead, they were more likely to talk about the effects of the armed conflict from generalizations or in third. This is paradoxical if we consider that most victims, in terms of deaths, of the armed conflict in Colombia are men [3].

### Mental health

The victims gave primary importance to mental health, placing it as a direct impact of the armed conflict since all physical effects also affect emotions, thoughts, and mental health. This was a topic addressed by both women and men. Participants never referred to "mental" health alone but also used the adjectives "psychological" and "emotional". The effects on these components were mentioned as something inherent to the armed conflict:

*Moderator:* *And speaking of all these things*, *how do you feel about your health*? *How do you perceive your state of health at this time*?

*Woman:* *When we look physically well*, *we are not in good health*, *starting with one of the factors that torment us the most*, *which is that psychological health or those emotional gaps that we have because of the armed conflict—and that they do not even see it as a disease*, *and it is a disease*. *That is what I can tell you today as a social worker*, *that now this is what is killing the victims of the armed conflict*. *(Mixed focus group in Castilla la Nueva*, *sept3)*

Those psychological effects, referred to as direct consequences of the conflict, were perceived as experiences that later become stress and physical symptoms [43]. They affirm that such psychological effects are sometimes irreversible and can condition people’s health in the long term. Furthermore, interventions focused on the struggle caused by the healing process of war wounds, were repetitive and did not have a remarkable outcome.

*Moderator:* *… did you want to say something*? *This health relationship affects you and your state of health today as related to the conflict*.

*Man:* *No* … *What she said*, *one does not enjoy that health because of what she said*, *to be well you have to be physically and mentally well*. *You can be physically well*, *sharp*, *nothing can be hurting you*, *but the mind can—because reiterating what she says*, *they are impossible to erase*. *One says*: *no*, *that eventually heals*, *but that’s a big lie*. *That happened to us ten years ago*, *and it is time*, *as I say*, *my children still remember*, *and you remember*. *I clearly remember what happened*, *the last time we had that problem*, *the time*, *the moment*. *I remember how they threatened us*, *the words that they told us*. *It is a lie that one is told that one is going to forget*. *The psychological damage afterward is irreversible and has no fix (Mixed focus group in Castilla la Nueva*, *sept3)*.

In numerous references, mental health was conceived as a component that influences structural aspects in people’s growth. If left unattended, it can have negative consequences on life trajectories. Most victims indicated emotional gaps left by the war due to the effect on relatives or the experience of events of direct violence. For example, those who have been stripped of their parents by the conflict reported emotional difficulties that are very difficult to remedy, coupled with fewer economic opportunities during their personal development. They also pointed towards consequences that these gaps have, such as bad attitudes in young people or aggressive or passive attitudes of victims who have not healed their wounds left by the war. Some drew attention to the need to deal with these traumas. In these same testimonies, it is pointed out that, as victims, they have not yet received the necessary emotional and psychological care because the impact of conflict in mental health tends to be trivialized or because it is a subject that is judged negatively.

### Barriers to the use of health services

The use of health services was discussed in depth in most focus groups, based on the participants’ personal experiences and their general knowledge of the health system. Most participants mentioned that there are several delays in using health services. This process was called "running" (*correrío*) or "paperwork" (*papeleo*) by some of the participants.

*Man:* *You go there and watch many people coming and going*, *but you don’t see a doctor there anymore*. *You see a few people with computers and screens*, *and "your turn is such and take documents*, *bring me this document*, *bring me this other*,*" until when finally*: *"Look*, *your appointment is set for three months from now"*. *In other words*, *you see a lot of people waiting for the system to give you the opportunity for the doctor to attend to you*. *(Focus group with men in Villavicencio*, *Sep2)*

Faced with these negative perceptions, several participants gave ideas about how the health system should be more human. It was proposed that instead of the common situation in which the doctor is behind a screen, doctors should inquire about the patient’s heart and make a better effort to know more things about people’s lives.

*Woman:* *(…) because there is a person behind a computer who is looking at a database saying "Oh*, *no*, *well this one has paid social security*, *this one doesn’t need it"* … *But does he know how the person’s heart is*? *So*, *maybe you may not need money*, *but you are in need of something else*, *another type of attention*, *yes*? *So*, *first of all*, *they need to know us*, *identify us*, *know what each of us feels*, *to spend a little time in our shoes*. *It’s not just sitting there on a computer to watch*. *(Focus group with women in Granada*, *Sept4)*

Some even referred to the fact that when the health system is assisting them, they feel discriminated against because they are victims:

*Man:* *(…) Well*, *in this hospital*, *I have not had so much inconvenience*. *When I was in the other hospital*, *a doctor said*: *"hey*, *the victims are given gifts*, *and they come angry to fight*.*" I said to her*: *"doctor*, *it is not that we are given things for free*, *we have a right to health*.*" I said to her*: *"no*, *it is not that we are asking for gifts; it is a right that we have*.*" So yes*, *reluctantly*, *she attended us*. *But you go to the hospital and just because of the fact because I have verified it*, *with the sole fact that they look at you as a victim*, *at once they make a face like "ugh*,*" right away*. *(Castilla la Nueva mixed focus group*, *Sept3)*

This discrimination was reported in Granada and Castilla La Nueva, municipalities that have been classified as having a light intensity of conflict and no conflict, respectively. Due to the lesser impact the conflict had on these communities, the recognition of the victims is less. However, as the participants stated, these municipalities are recipients of victims who have been displaced from other places. Some victims considered that the difficulties they experience when trying to use health services represent another victimization process. These difficulties in receiving care were accompanied by critical visions about the operation of the health system: delays in care are intentional; not enough information is provided through which entities promote their services; the system leads the doctors themselves not to provide adequate care; and, despite the lack of capacity, healthcare provider organizations continue to enroll more and more beneficiaries into their care systems.

Against this background, many prefer not to go to the health system to solve their health problems, and they resort to other options such as self-medication, going to a private doctor, or not treating the disease. Self-medication is one of the most frequent strategies as an alternative to attending health services. This is due to the word-of-mouth that runs between people. There is a certain knowledge about which medicine people should take depending on the symptomatology. Similarly, on two occasions, the participants mentioned that they searched on the internet to see what medicine they can take or what foods to eat according to their illnesses. The option of attending a private medical consultation was especially justified when urgent or necessary care is required. There was also an appeal to take up ancestral knowledge about plants as an alternative to health services, where care is not always provided satisfactorily. Finally, two women stated that they required the medical services of illegal armed groups to save the lives of their children at certain times.

The difficulties in using health services are more pronounced in people from areas further away from health service specialties, such as in Castilla la Nueva or Vistahermosa. People prefer other types of solutions in those municipalities instead of wasting time and energy looking for solutions in health care services.

*Woman:* *You start the process here in Castilla la Nueva*. *The staff provide care with little equipment and with a few basic doctors*. *Then they send you to Granada or Villavicencio*. *There you begin a process in which they give you acetaminophen and send you home*, *even if you are unwell*. *Then*, *for example*, *Capital Salud takes between 15 and 20 days to authorize the exam*, *then to schedule that exam*, *and then to be attended again (…)*, *But you start with a process that is fatal*. *So*, *sometimes we go through the process*, *but we know that the best thing is to go to the drugstore and buy a pill*. *It’s better than a queue of 4 hours (Mixed focus group in Castilla la Nueva*, *sept3)*

In the focus groups that were held in these municipalities, it was mentioned that some NGOs provided extramural health services and in remote villages before the peace deal. The most mentioned was *Médicos del Mundo*, which would travel to rural areas where there were severe security conditions to provide urgent and preventive medical care. However, this NGO would provide attention to the population "during the conflict," and after the Peace Agreement, they stopped providing services. The participants of Vistahermosa and Castilla La Nueva have very positive memories and perceptions of the work carried out by *Médicos del Mundo*. This NGO provided essential services such as dentistry, pediatrics, and gynecology. Likewise, they provided care to those wounded in combat, regardless of whether patients were civilians or combatants. Thus, participants felt that since the accord, healthcare quality had decreased.

### Specialized care for victims: Is there any?

One of the emergent topics that was discussed in some focus groups was the mechanisms of specialized care for victims. In this regard, there were diverse experiences. While some never received specialized psychological attention, others indicated a lack of continuity of the victim’s program, *Programa de Atención Psicosocial y Salud Integral a las Victimas* (PAPSIVI). What everyone agreed on was the need for differential and timely care for the victims of the conflict.

*Woman:* *For all these reasons*, *care should be different*.

*Moderator:* *Why should it be different*, *and why is it not*?

*Woman:* *First of all*, *because our rights have been violated*. *As victims*, *we should have had first aid*, *but we did not have it*. *We were displaced*, *and where did we have to go*? *Some sleep in the park*, *others in this booth*, *and others there*. *And some disappeared*, *others were killed*. *And we don’t get first aid*. *(Focus group women Vista Hermosa Sept5)*

### Has anything changed since the peace agreement regarding health?

The participants’ overall perception was that "nothing has changed" in terms of health services after the peace agreement. The difficulties in receiving medical attention result mainly from structural issues rather than from the conflict itself. Participants who noticed positive changes pointed out that in some places where there was a previous presence of armed groups, it is possible to travel more easily, allowing greater mobility for health services. However, they felt that the agreements of peace accord have not been fully complied with and does not prevent the conflict from resuming:

*Man:* *(…) But the state has never really been present*. *The state remained on the issue of peace*, *but the health issue was left abandoned*, *and it is something that has not improved*. *The theme of peace*, *for the territory of Vistahermosa I say super awesome*, *we can live well and calm*. *We no longer have to go to bed early and look at what time we wake up*, *like we did before when there were shootings*. *Now we can walk all night if we want through the territory without any problem*. *But really*, *the benefits that we thought could come and that were in the Havana agreements*, *those things have not been seen*. *(Focus group with men in Vistahermosa*, *Sept5)*

In Vistahermosa, despite having been the municipality most affected by violence, they were emphatic in blaming the state and not the conflict itself for the health system’s problems.

Similarly, when inquiring about the effects of the armed conflict on the health system, there were different positions. For some, the conflict did affect the health system. These participants pointed to some facts, for example, damage to infrastructure, restrictions on mobility, and lack of guarantees for health professionals’ work. In contrast, others insisted that the main reason for the system problems was mismanagement. They argued that the same system is conditioned by structural aspects, such as health providers’ institutions’ problems. In this regard, participants mentioned the detriment of the health providers’ institutions that have gone bankrupt, collapsed, or experienced deteriorating attention. This is reflected in a lack of medical attention, specifically for patients in the subsidized system. Thus, there is a generalized negative perception regarding the health system, which is affected by other structural factors such as conflict, lack of infrastructure, and the health system model itself.

## Discussion

Through focus groups discussions and subsequent analysis, we found that the most pressing aspect for victims’ resident in post-conflict areas is stigmatization and revictimization that stem from the armed conflict itself, and from a historical lack of structural social and health care services. Victim´s experiences obtaining timely and appropriate health care, three years after the peace agreement highlight the effect of conflict on mental health as a prevalent issue among children, women and men. Likewise, it reflects the need to understand the barriers that the health model imposes on the right to health itself. Victims perceive and report stigmatization and discrimination given their victim status plus the direct effects of the armed conflict. Revictimization experiences result when victims have to recount multiple times their experiences to justify the need for health services or in the process of doing so for administrative purposes. These insights contribute to understanding bottlenecks for victims of armed conflict when accessing health services and not shared with other vulnerable or violence-affected populations within the country.

The focus groups promoted a performative ritual of activating certain narratives [35] in the space of dialogue between victims of the armed conflict. The victims who participated in the focus groups accounted for the painful effects of the conflict and supported civil actions by citizens, anchored in their position as victims [30, 33].

The ideas that participants shared about health demonstrate how victims perceive their health and the tensions in the context of a precarious offering of services. These notions result from intersubjective productions from a common collective imagination and constant feedback with the public sphere [38]. In this research, women’s experience of conflict, and the effects of conflict on mental health, constitute central themes in the intersubjective dialogue of victims. These were the elements with the greatest narrative visibility, as they emerged recurrently from the connection between group narratives about the conflict and perceived health impacts [36].

On the topic of women’s experience of conflict, the fact that in the focus groups, women were more open to talking about their experiences of violence during the conflict, may be related to the fact that more women participated and may have been relatively more vulnerable during wars and faced increased risks of sexual and gender-based violence and forced displacement [44]. To the extent that violence is a common factor in patriarchal societies, women face significant challenges to their health in post-conflict contexts [45]. As victims of sexual abuse and violence that increase during war and peace processes, women may be motivated to fight for peace more frequently than men because they want to protect themselves, their children, and their communities from these types of violence. Thus, women who share their experiences of violence do so from the active participation in the reconstruction of the social fabric, even more, when sexual violence has been used as a war tactic to dominate, exterminate, silence, or punish [46]. Our participants had experience with victims’ organizations, which may explain these political/leadership perceptions and recounts of their experiences.

The topic of mental health was addressed by both men and women, from personal experiences and their families. Mental health is an issue that victims increasingly demand. In the focus groups, this was considered a direct effect of the armed conflict since all its effects on people’s physical health occur simultaneously with effects on people’s emotions, thoughts, and states of mind. The effects on mental health were reported as something inherent to the armed conflict that is expressed to a higher or lower degree depending on the proximity to the conflict. Indeed, the need to advance comprehensive psychosocial care for all victims of the internal armed conflict in Colombia has already been widely recognized to respond to emotional suffering and break the cycles of violence that culminate in poly-victimization [47]. Thus, it is important to consider these perceptions to recognize people’s personal and social history in care and treatment settings [48].

The description of the participants’ physical and psychological effects on health was not exclusive to violent experiences; it was also related to continued difficulties in receiving adequate medical attention. By becoming a victim, the person begins a tortuous path to seek social, physical, mental, and economic reparation. Moreover, if that search does not end in the provision of effective care, its conditions tend to worsen. For the victims, this tortuous path is because going to the health system often becomes what the participants called "running" or "paperwork," and it is impossible to receive adequate care from health professionals. Participants spoke about the need of adequate care; referring to the relationship between the doctor and the patient should be more empathic. In their words, instead of the usual situation in which the doctor speaks while looking at a computer screen, he/she can inquire about how the person/patient is feeling.

These perceptions appeal to difficulties that all Colombians affiliated to the subsidized regime have to face. This has already been described by Abadía [6] as "bureaucratic itineraries", which shows how these constitute barriers that the system itself has to ensure patients’ rights to health. Some of them are administrative problems with the system, financial barriers, institutional deficiencies in human or physical resources, negligence, and limited policy coverage problems. These barriers have negative consequences for people’s lives, evidenced in prolonged suffering, medical complications, disabilities, and death. It is important to see if these barriers affect victims in the same way or any different factor that has to be considered for this population. In this sense, Cardona, Veloza, & Lopez [23] propose that access to health services by victims of the armed conflict involves almost the same problems and difficulties in access and quality of services that most Colombians have to face.

Participants also mentioned experiences and perceptions of stigmatization and revictimization when using health services, which has led to even greater hopelessness among victims. This imposes an additional burden on these people’s psychological well-being and represents a barrier to access health services even when the need for services is entirely manifest [49]. This situation highlights the difficulties that victims have in particular to make use of health services for several decades [50]. Specialized health attention for victims, while for some is non-existent, for others is available but they point that there is a lack of continuity or undesired results. They agreed that there should be specialized victim care that is comprehensive and inclusive so that the life trajectories of the victims and their families are considered. Previous studies have shown that the impacts of armed conflict on physical, mental, and behavioral health in children can persist throughout the entire life cycle [43].

Health institutions and service providers require tools to assist the victim population in ways that access and care are understood beyond individual one-off encounters in addition to the clamor for justice that this population makes to Colombian society [26]. In this sense, the decision to speak openly about the experiences of violence, while having a dimension that has to do with the still-pending healing of wounds, also has a political background to the extent that the person willingly decides to talk about their experiences of violence, compared to others who have also suffered because of the conflict. The fact that women have talked about their experiences of sexual violence indicates that there is still a need for them to be heard for multiple reasons, including healing, reporting, and reparation, which can be considered as their demands of victims as political subjects. Hence it is also important to insist that the mere condition of having experienced a traumatic event is not equivalent to label him/her as a victim but as someone who can find reparation in the context of the process in which other victims are located. In Colombia, this process has had such a development that a victim means a collective subject who has had important achievements such as the Victims Act itself.

The priority care for victims of the armed conflict is established by Law of Victims 1448 of 2011, which established the PAPSIVI as a program of social and psychological support for this population, program that is still in force. This program is based on human rights principles, public health, and community psychology. It aims to increase access to mental health services for victims of the conflict through community doctors, psychologists, and social workers. However, this program has not been managed to have significant coverage, and concerns have been raised about its slow implementation and the limited capacity to provide services to millions of people [51]. Thus, despite the existence of regulations, plans, projects, and programs that regulate and provide guidelines for the care of victims of the conflict, this population’s care does not evidence differential or comprehensive care [15].

### Strengths and limitations

Several strengths and limitations were identified. Focus group discussions allowed participants to share in a safe space their experiences in profound ways contributing to our understanding of their health perceptions and the problems faced by victims for future studies, interventions, and health and post-accord decision-makers. Our recruitment strategy through local organizations for victims had the strength of securing trustworthy channels of communication with victims in the study setting, and this allowed us to have focus group activities in all the proposed municipalities despite the sensibility of the topic of study. Among participants, more women than men attended our focus groups, and we were not able to recruit men in one municipality. Some reasons for the lower participation of men could be explained by active conflict resurgence during our period of fieldwork, overlapping local meetings where men actively participate, and collective perceptions of hopelessness on the possibility of change. In addition, from our qualitative research experience in Colombia, women are more likely to participate when the discussions are about health given their cultural role of caregivers of the family and relatively higher demand for health services because of childcare, maternal needs, and care for the elderly. The qualitative nature of our study may not allow for the generalization of our findings to other post-accord settings in other cultural and political contexts.

## Conclusion

Based on the relationship between trauma, health, and victim as a political category, this research calls attention to the issues that emerged from the victims’ intersubjective dialogue: mental health and gender violence. It also highlights the additional pressures victims of the armed conflict have on their health and access to health services. This is important because political violence is just one more factor that adds to poverty and social marginalization [52], such as that experienced by the territories most affected by the armed conflict in Colombia. Thus, the results of this research can be considered in the process of reparation of victims in the Colombian post-conflict context, as it points to the political relevance of the victims’ demands regarding the right to health and some of the daily stress factors they face.

Understanding how the victims’ situation has changed after the Peace Agreement is an essential step to grasp the challenges that persist concerning this population. Reparation processes should identify and overcome bureaucratic barriers to prevent revictimization cycles that impede access to health services and affect the victims’ rights to psychosocial reparation [53, 54]. Also, health workers can better approach restoring victims’ civil rights based on local knowledge of populations affected by the conflict.

## Supporting information

S1 Text Informed consent and study information. (DOCX)

S2 Text Focus group guide questions. (DOCX)
