# Supplementary material for: Health perceptions among victims in post-accord Colombia: Focus groups in a province affected by the armed conflict
Source: PLoS One. 2022 Mar 2;17(3):e0264684. doi: 10.1371/journal.pone.0264684 (PMC8890648; doi:10.1371/journal.pone.0264684)
Supplement: S1 Text — (DOCX) [file pone.0264684.s001.docx]

**Informed Consent and Study Information**

*War and Peace*

Introduction of researchers:

Good morning, my name is _________________________ and I am ___________________-

This project seeks to understand the effects of armed conflict on people´s health, as well as on the health system, and also to provide public policy recommendations to address the health challenges in the post-accord era. The information you give us during this process will contribute to the construction of one or more publications in which it will seek to respond what the effects of the conflict have been on the health of Colombians, how the health system has been changing and adapting to the conflict, what are the health challenges in the post-agreement era, as well as in the design of public policy recommendations to overcome these challenges. This research project is part of the Alberto Lleras Camargo School of Government at the University of the Andes and York University in England.

Description of Activities:

If you decide to collaborate with this project, you will participate in qualitative data collection activities including the following individual and/or group activities:

•         Answer general questions about your perception of the topics mentioned above.

•         Participate in group conversations

•         Participating in social mapping activities

Risks:

Some of the questions can cover topics that are uncomfortable for you to answer, it is not mandatory to answer all questions, the main objective is to generate a comfortable space for you as well as for the researcher.

Benefits:

This study is not designed to help you directly, but what we learn can help others in the future and help us build an action plan to include your voices and opinions in post-conflict health action and promotion processes in Colombia.

Confidentiality:

Everything we learn from you in this study is confidential. At no time will your name or identity be disclosed, nor will the individual data be disclosed, as these will be used only by the team and will be used anonymously in academic writings.

Compensation:

For data collection activities, you will receive compensation equivalent to your transportation costs (round trip) for your residence premises.

Voluntary Participation:

You are not obligated to participate in the study and no one will bother with you if you choose not to participate, or if you decide to withdraw from the study at any time for reasons that you are not required to clarify. If you decide to withdraw, you will still receive compensation equivalent to your transportation costs.

If you have any questions about the study, you can call Oscar Bernal at 3394949 Ext. 4725 or write an email to obernal@uniandes.edu.co and/or Sebastián León Giraldo at 3016065164 or write to email sd.leon10@uniandes.edu.co.

In case of any problems associated with the research, you can also contact the Ethics Committee of the University of the Andes. Phone 3394949 Ext. 5339 or email [comite-etica-investigaciones@uniandes.edu.co](mailto:comite-etica-investigaciones@uniandes.edu.co) .

*Would you like to participate in the study?*Yes _____ No______

*Can we record activities in audio and/or video?*Yes _____ No______

__________________________                   ____________

*Participant's Signature Date*

__________________________                   ____________

*Signature of Witness 1  Date*

__________________________                   ____________

*Signature of Witness 2  Date*
