# Supplementary material for: Health perceptions among victims in post-accord Colombia: Focus groups in a province affected by the armed conflict
Source: PLoS One. 2022 Mar 2;17(3):e0264684. doi: 10.1371/journal.pone.0264684 (PMC8890648; doi:10.1371/journal.pone.0264684)
Supplement: S2 Text — (DOCX) [file pone.0264684.s002.docx]

**Focus group guide questions**

**Group time:**about 1 hour

*Instruction:*Sort and write down the ideas that the group participants are mentioning around the categories as if they were clouds of words. Icebreaker activity is going to be the first two health questions.

1. What is health for you?

2. What is disease?

3. How do you perceive your health?

4. How do you rate the health of people living in your municipality?

5. What are the mos commom health problems? (be aware of differences by gender)

6. Where do you go or what do you do when you get sick?

7. Who is the caregiver in your home?

*Conflict*

1. Do you think armed conflict has affected the provision of health services?

2. What changes do you see before and after the signing of the Peace Agreement?

3. Regarding to your health, what were you worried about 10 years ago?

4. What did you do in conflict when you got sick or had a health-related problem?

5. Do you think there are any health problems related to the history of conflict in the municipality?
